# The association between adverse experiences throughout the life‐course and risk of dementia in the English Longitudinal Study of Ageing

**DOI:** 10.1002/alz70860_100960

**Published:** 2025-12-23

**Authors:** Katherine Taylor, Laura Howe, Rebecca E Lacey, David J Carslake, Emma L Anderson, Naheed Mukadam

**Affiliations:** ^1^ University College London, London, London, United Kingdom; ^2^ University of Bristol, Bristol, Bristol, United Kingdom; ^3^ St Georges, University of London, London, London, United Kingdom; ^4^ Camden and Islington NHS Foundation Trust, London, London, United Kingdom

## Abstract

**Background:**

Previous studies on the association between adverse experiences and dementia focus on a narrow range of experiences and use unrepresentative samples, often with short follow‐up periods and limited dementia cases. Additionally, most use cumulative measures of trauma, which assume each experience has the same impact on dementia risk. We aimed to examine both cumulative and categorical measures of adverse life experiences in a sample more representative of the UK population to better elucidate which experiences drive associations between adverse experiences and dementia.

**Method:**

The English Longitudinal Study of Ageing measured adverse experiences in a retrospective life history interview. We first considered experiences singly, then grouped with other similar experiences into broad adversity measures. We also generated total count measures. We used logistic regression to investigate the association between total count measures and dementia incidence, estimating two models, one assuming that no censored participants developed dementia, and one assuming that all censored participants developed dementia. The true association should lie somewhere on a spectrum between the results from the two. Cox proportional hazard models were used to investigate hazard of dementia in association with, singular, broad and total count categorisations of adversity. We also examined effect modification by sex and childhood economic hardship.

**Result:**

Overall, there was little evidence that total count measures of adversity throughout life were associated with dementia incidence. Experience of child abuse was associated with a 75% increased hazard of developing dementia (HR: 1.75, 95% CI1.25‐2.44) and severe adult economic hardship was associated with a 32% increased hazard of dementia (HR:1.32, 95% CI 1.06‐1.66). Adult household challenges were associated with dementia but only among those who experienced severe economic hardship in childhood (HR:2.99, 95% CI: 1.48‐6.06).

**Conclusion:**

Experience of child abuse or severe economic hardship in adulthood increases an individual's risk of dementia. Use of additive approaches to operationalise adverse experiences may oversimplify associations between adverse experiences and dementia. Our results suggest certain adverse life events increase risk of dementia and further work should address modifiability of these exposures.

